# Late-stage rescue of visually guided behavior in the context of a significantly remodeled retinitis pigmentosa mouse model

**DOI:** 10.1007/s00018-022-04161-0

**Published:** 2022-02-23

**Authors:** Jacqueline Kajtna, Stephen H. Tsang, Susanne F. Koch

**Affiliations:** 1 Department of Pharmacy, Center for Drug Research, Ludwig-Maximilians-Universität München, Munich, Germany; 2 Physiological Genomics, BioMedical Center, Ludwig-Maximilians-Universität München, Planegg/Martinsried, Germany; 3 Jonas Children’s Vision Care, Columbia Stem Cell Initiative, Departments of Ophthalmology, Pathology and Cell Biology, Institute of Human Nutrition, Vagelos College of Physicians and Surgeons, Columbia University, New York, NY 10032 USA; 4 Edward S. Harkness Eye Institute, New York-Presbyterian Hospital, New York, NY 10032 USA

**Keywords:** Retinal degeneration, Gene therapy, Remodeling, Retinitis pigmentosa, RPE, Retinal blood vessels

## Abstract

Patients with progressive neurodegenerative disorder retinitis pigmentosa (RP) are diagnosed in the midst of ongoing retinal degeneration and remodeling. Here, we used a *Pde6b*-deficient RP gene therapy mouse model to test whether treatment at late disease stages can halt photoreceptor degeneration and degradative remodeling, while sustaining constructive remodeling and restoring function. We demonstrated that when fewer than 13% of rods remain, our genetic rescue halts photoreceptor degeneration, electroretinography (ERG) functional decline and inner retinal remodeling. In addition, in a water maze test, the performance of mice treated at 16 weeks of age or earlier was indistinguishable from wild type. In contrast, no efficacy was apparent in mice treated at 24 weeks of age, suggesting the photoreceptors had reached a point of no return. Further, remodeling in the retinal pigment epithelium (RPE) and retinal vasculature was not halted at 16 or 24 weeks of age, although there appeared to be some slowing of blood vessel degradation. These data suggest a novel working model in which restoration of clinically significant visual function requires only modest threshold numbers of resilient photoreceptors, halting of destructive remodeling and sustained constructive remodeling. These novel findings define the potential and limitations of RP treatment and suggest possible nonphotoreceptor targets for gene therapy optimization.

**Supplementary Information:**

The online version contains supplementary material available at 10.1007/s00018-022-04161-0.

## Introduction

When patients with degenerative diseases are diagnosed, they have usually already suffered irreversible cell loss [1]. The goal of gene therapy, therefore, is ultimately to halt further degeneration, even at late disease stages, to retain some function. This is certainly the case with retinitis pigmentosa (RP), an inherited progressive blinding disease driven, most often, by dysfunction of rod photoreceptor cells that leads to their death, and secondarily, the death of cone photoreceptors [2]. In an RP mouse model, we have shown that gene therapy administered at middisease stages stops further photoreceptor loss and progressive degradation in retinal function [3]. However, it is not known whether gene therapy restores vision at late disease stages, when the vast majority of photoreceptors (rods and cones) have degenerated.

We know from studies in mice that progressive changes in RP retina manifest beyond the photoreceptors—in, for example, the retinal pigment epithelium (RPE) [4], the retinal vasculature [5] and the downstream neurons of the inner retina [6]. The inner retinal neurons, especially the rod bipolar cell dendrites and horizontal cell processes, remodel extensively [7–9]. This disease-driven remodeling has been described to be either maladaptive/degradative or constructive. It has been suggested that degradative remodeling can exacerbate disease and impede RP treatment strategies [10–12]. In other studies, data suggest that constructive remodeling can compensate functional loss [13, 14].

Human gene therapy trials for an inherited retinal disorder reported temporary restoration of some visual function [15–18], suggesting that of the remaining diseased photoreceptors, a critical number had reached a point of no return [19]. It is unclear if gene therapy impacts constructive remodeling and, if it does, whether this drives some of the restoration of visual function.

In this study, we used an RP gene therapy mouse, in which the floxed stop cassette in intron 1 of the gene encoding rod-specific phosphodiesterase 6b (*Pde6b*) can be removed using Cre. Our Cre/loxP genetic rescue approach greatly facilitates the analysis of gene therapy efficacy in comparison to surgical application of viral vectors. It overcomes the negative aspects usually associated with subretinal injections (e.g., trauma, retinal detachment and limited number of treated photoreceptors) and decreases the between-injection variability while providing optimal *Pde6b* gene transcript levels. Mutations in *PDE6B* are a common cause of autosomal recessive RP in humans [20]. We first used these mice to test whether photoreceptor degeneration can be halted even at late disease stages and the degree to which the treatment restores both photoreceptor function and visually guided behaviors. We also used these RP mice to study retinal remodeling during disease progression and to test whether this remodeling is affected by genetic rescue.

## Materials and methods

### Animals

All animal experiments were performed according to the ARVO statement for the use of animals in ophthalmic and vision research and were approved by the local authorities (Regierung von Oberbayern). Mice were kept under standard conditions under a 12-h light/12-h dark cycle with access to water and food ad libitum*.*

*Pde6g*^*CreERT2*^ and *Pde6b*^*STOP*^ mice were generated in the Barbara & Donald Jonas Stem Cells Laboratory, Columbia University, USA [3, 21, 22]. A stop cassette was inserted in intron 1 of the *Pde6b* gene. In *Pde6g* exons 2 and 3 were replaced by tamoxifen-inducible CreERT2. Mice were rederived via in vitro fertilization at the Biomedical Center Munich, Germany. In our study we used mutant (*Pde6b*^*STOP/STOP*^, *Pde6g*^*CreERT2/*+^*;* referred to as *Pde6b*^*STOP/STOP*^) and control mice (*Pde6b*^*STOP/*+^, *Pde6g*^*CreERT2/*+^; WT) of both sexes. Primers and their annealing temperatures used for the genotyping are given in Table 1. Some mice were also genotyped for absence of spontaneous mutations rd1, rd8 and cpfl3.

**Table 1:** Primers for genotyping

Target	Forward primer (5′ → 3′)	Reverse primer (5′ → 3′)	Internal primer (5′ → 3′)	Annealing temperature (°C)
*Pde6b*^*Stop*^	TGCTCTGTGGTGTTGCTCTGC	TGGCGATGCAGAGTGTCCTGA	GTCCTGCACGACGCGAGCTG	65
*Pde6g*^*CreERT2*^	GGTCAGATTCCAGTGTGTGGG	GTTTAGCTGGCCCAAATGTTG	CTTAGGTGGTCCTTTCCTGGG	65

### Tamoxifen treatment

Tamoxifen (#T5648, Sigma-Aldrich) was dissolved in absolute ethanol to a concentration of 100 mg/mL, sonicated for 15 min, and further diluted in corn oil to a stock solution of 10 mg/mL. It was administered intraperitoneally at a concentration of 100 µg/g body weight on two consecutive days (1 injection/day).

### Tissue preparation and immunohistochemistry

Mice were euthanized, and the temporal side of their eyes was marked. Eyes were enucleated, the anterior segments and vitreous were removed and eyecups were fixed in 4% paraformaldehyde (PFA) in phosphate-buffered saline (PBS), pH 7.4 for 45 min.

For cryosections, eyecups were washed three times in PBS (pH 7.4), cryoprotected overnight in 30% sucrose, embedded in Tissue-Tek® O.C.T.™ compound (Sakura) and frozen. Eyecups were then sectioned vertically at 10 µm using a Leica cryostat, collected on Thermo Scientific™ SuperFrost Plus™ slides and stored at – 20 °C. For staining, retinal sections were thawed, washed with PBS and incubated overnight at 4 °C in primary antibodies (Table 2) in PBS containing 5% Chemiblocker (#2170, MerckMillipore) and 0.3% Triton® X-100. Subsequently, sections were washed in PBS (3x) and incubated with secondary antibodies (Table 2) in PBS containing 3% Chemiblocker for 2 h at room temperature. For nuclear counterstaining, sections were incubated for 5 min in 5 µg/ml Hoechst 33342 (#H1399, Invitrogen), then washed again with PBS and mounted with Aqua-Poly/Mount (Polysciences) medium.

**Table 2:** Primary and secondary antibodies/ lectin

Antibody/lectin	Host species	Dilution	Manufacturer	Catalog number	Conjugate for secondary antibodies
Pde6b	Rabbit	1:4000	Thermo Fisher	PA1-722	
Cone arrestin	Rabbit	1:1000	Merck	AB15282	
Secretagogin	Rabbit	1:5000	Generous gift from Prof. Dr. Ludwig Wagner (Univ. of Vienna, Austria)	[23]	
Rhodopsin (1D4)	Mouse	1:1000	Santa Cruz	sc-57432	
PKCa (H-7)	Mouse	1:1000	Santa Cruz	sc-8393	
Calbindin D-28 k	Mouse	1:8000	Swant	300	
ß-catenin	Rabbit	1:500	Cell Signaling Technology	8480S	
Isolectin B4 conjugated FITC		1:100	Sigma-Aldrich/ Merck	L2895	
488-Goat anti-rabbit	Goat	1:1000	Thermo Fisher	A-11070	Alexa Fluor 488
647-Goat anti-rabbit	Goat	1:1000	Thermo Fisher	A-21245	Alexa Fluor 647
555-Goat anti-mouse	Goat	1:1000	Thermo Fisher	A-21425	Alexa Fluor 555
647-Goat anti-mouse	Goat	1:1000	Thermo Fisher	A-21235	Alexa Fluor 647

For flat-mounted retinal and RPE–choroid–sclera preparations, eyecups were washed in PBS (3x). The retina was removed from the RPE–choroid–sclera. The RPE–choroid–sclera preparations were bleached in 10% H_2_O_2_ at 55 °C for 1.5 h. The retinal and RPE–choroid–sclera flat mounts were incubated overnight at 4 °C in primary antibodies (Table 2) in PBS containing 3% DMSO, 0.5% Triton X-100 and 5% Chemiblocker. Subsequently, the flat mounts were washed with PBS and incubated for 2 h at room temperature with secondary antibodies (Table 2) in PBS containing 3% Chemiblocker. For nuclear counterstaining, flat mounts were incubated for 10 min with Hoechst 33342, then washed again with PBS and mounted with Aqua-Poly/Mount medium on slides.

Samples were imaged with laser-scanning confocal Zeiss LSM710, Leica TCS SP8 or custom-made VisiScope CSU-X1 confocal system equipped with a high-resolution sCMOS camera (Visitron Systems, Puchheim, Germany).

### RPE morphometry

RPE–choroid–sclera flat mounts were stained for ß-catenin. Images were acquired with a confocal microscope using 40 × oil objective in three regions of the flat mount: central, equatorial, and peripheral [24]. After adjusting contrast and brightness in ImageJ, areas of equal size were used for the automatic morphometric measurements in CellProfiler [25]. Not accurately segmented individual RPE cells were manually excluded from the analysis. The values for the three morphometric parameters of the RPE cells from each image were distributed over selected bins and the mean (± SEM) percent of the RPE for each group was plotted. The *N* numbers are given in Table 3.

**Table 3:** Number of mice for the RPE morphometric analysis

Region\group	Mutant pw16	Mutant pw24	Mutant pw40	Treated pw16/40	Treated pw24/40	WT pw40
Central	5	5	5	5	6	6
Equatorial	6	5	5	4	6	5
Peripheral	5	6	8	4	5	5

### Retinal vessel area quantification

Retinal flat mounts labeled with isolectin GS-IB4-conjugated FITC were scanned in z‐stack mode (1 µm steps) from the top to the bottom layer in up to three central and peripheral regions and merged in ImageJ. AngioTool software was used to quantify the percent vessel density (University of Warwick, UK) [26].

### Trypsin digestion and H&E staining

Retinas were digested using trypsin as previously described with slight modifications [27]. Briefly, after enucleation the eyecup was fixed in 4% PFA for 45 min, then the retina was isolated and further fixed in 4% PFA overnight. The next day, the retinas were washed (5x) with distilled water. After overnight shaking in water at room temperature, retinas were transferred into 24-well plates and incubated in 3% trypsin (#9002-07-7, Affymetrix USB, Ohio, USA) in 0.1 M Tris buffer (pH 7.8) at 37 °C for 90 min. The inner limiting membrane was removed with scissors and then the vasculature isolated by several washing steps. The retinal vasculature system was flat mounted on Thermo Scientific™ SuperFrost Plus™ slides and stained with H&E (H&E fast staining kit, #9194.1, Carl Roth). Microscopy was performed at the Core Facility Bioimaging of the Biomedical Center at the LMU with a Leica DM6 FS microscope. Bright field images were recorded with Leica DMC2900 CMOS camera with an image pixel size of 145 nm. A 20x/0.8 objective was used for quantification and overview image of the network and a 40x/0.95 objective for detailed view of the vascular network.

### Quantitative analysis of acellular capillaries

Acellular capillaries were manually counted using ImageJ software. For each animal, up to five areas of 0.06 mm^2^ each were counted. Acellular capillaries were detected as vessel tubes without nuclei. *N* numbers for WT mice were 3, 6, 4, and 9 at the age of 16, 24, 30, and 40 weeks, respectively. *N* numbers for mutant mice were 4, 6, 2, and 7 at the age of 16, 24, 30, and 40 weeks, respectively. *N* numbers for 40-week-old mice treated at 12, 16, or 24 weeks were 5, 3, and 4, respectively.

### Quantitative analyses of ONL thickness and rod photoreceptor number

The retinal cryosections were stained against cone arrestin and counterstained with Hoechst 33342. Images were taken in the ventral area of the retina (3 sections per eye). Using ImageJ, the ONL thickness was measured 250 µm from the optic nerve. The *N* numbers for mutant retinas were 4, 6, 6, 4, 7, 3, and 8 for 4-, 8-, 12-, 16-, 24-, 30-, and 40-week-old mice, respectively. The *N* numbers for WT retinas were 3, 4, 3, 6, 2, 3, and 8 for 4-, 8-, 12-, 16-, 24-, 30-, and 40-week-old mice, respectively. The *N* numbers for 40-week-old mice treated at 4, 12, 16 and 24 weeks were 4, 4, 3, and 3, respectively. ONL thickness values of mutant animals were fit to a one-phase exponential decay model [28]:

$$Y=y_o*e^{(-k+X)}$$

Rod numbers were determined by counting Hoechst-labeled nuclei in the ONL and subtracting the number of cones (i.e., arrestin-positive cells). Photoreceptors were counted in mutant retinas at 12, 16, 24, and 40 weeks of age (*N* = 5, 5, 4, and 4, respectively). Photoreceptors were counted in treated retinas at 12, 16 and 24 weeks of age (*N* = 5, 4 and 4, respectively). The rod photoreceptor number was normalized to 40-week-old WT mice (*N* = 4, rod number: 210.4 ± 5.9).

### ERG

Animals were dark adapted overnight, and all procedures were carried out under dim red light (used red filter: Rosco Supergel 27, Medium Red, #10273) [29]. Mice were anesthetized by intraperitoneal injection of 0.1 mL/10 g body weight of anesthetic solution (1 mL of 100 mg/mL ketamine, 0.1 mL of 20 mg/mL xylazine, and 8.9 mL of PBS). Pupils were dilated by one drop of 0.5% tropicamide (Mydriaticum Stulln 0.5%, Pharma Stulln GmbH) and 5% phenylephrine hydrochloride (Neosynephrin-POS 5%, URSAPHARM Arzneimittel GmbH). During measurements, corneal hydration was ensured by application of hypromellose (Methocel® 2%, OmniVision GmbH), and golden loop electrodes were placed on each cornea. Body temperature was maintained at 37 °C using a heating pad. ERG responses were recorded simultaneously from both eyes using an Espion E3 console in conjunction with the Color Dome. Scotopic electroretinograms were recorded at white light flash intensities of – 3, – 2, – 1.5, – 1.0, 0.5 and 1.0 log (cd*s/m^2^) (except for mice treated at 12 weeks, where intensities were −3 and 0.5 log (cd*s/m^2^)). For photopic measurements, mice were adapted for 10 min to a background of white light at an intensity of 30 cd/m^2^ to suppress the rods, and then recordings were continued at white light intensities of – 0.5, 0, 0.5, 1 and 1.5 log (cd*s/m^2^) (except for the for mice treated at 12 weeks, where the measurements were performed at intensities of 1.5 log (cd*s/m^2^)). The a-wave amplitudes were measured from the baseline to the peak of the negative a-wave (baseline to trough), and b-wave amplitudes were measured from the trough of the a-wave to the peak of the positive b-wave. For each animal, the mean response of both eyes was averaged. Data were analyzed using a two-way analysis of variance (ANOVA).

### Morris water maze

The Morris water maze task was conducted to assess visual function under dim (rod-dependent) light conditions (5.54 mW/cm^2^ at 508 nm at the water level; measured with Nova II laser power/energy monitor and the PD300 photodiode head, Ophir Optronics LD). The circular pool had a diameter of 160 cm and was filled with 24–26 °C warm water. The circular platform of 10 cm in diameter was submerged approximately 1.5 cm under water in a fixed position in the northern quadrant. The pool was surrounded by distal cues in the room. Mice were tested in eight trials per day for five consecutive days, with the starting position changed for each trial. For each trial, the mouse was gently inserted into the water facing the wall. The mouse was given 60 s to find the platform. If the mouse failed to find the platform within 60 s, it was gently guided to the platform with a stick. Mice were allowed to remain 15 s on the platform before being returned to their home cage. On the fifth day (after the last trial), the escape platform was removed from the pool, and the time spent in each quadrant during a single 60 s trial was measured (probe trial). Animals were tracked using the VideoMot2 software (TSE, Germany). Representative paths were converted from raster to vector with CorelDRAW.

### Statistics

Data are expressed as mean ± standard error of mean (SEM). The *N* values refer to the number of individual animals. All data were analyzed in GraphPad Prism 5.0 software (La Jolla, CA, USA), using two-way analysis of variance; *P* ≤ 0.05 was considered statistically significant (*P ≤ 0.05; **P ≤ 0.01; ***P ≤ 0.001). To compare multiple groups, we used Tukey's multiple comparison test and reported adjusted *P* values.

## Results

### Photoreceptor degeneration halted by treatment at 16 weeks of age or earlier, but not at 24 weeks

**Fig. 1:**
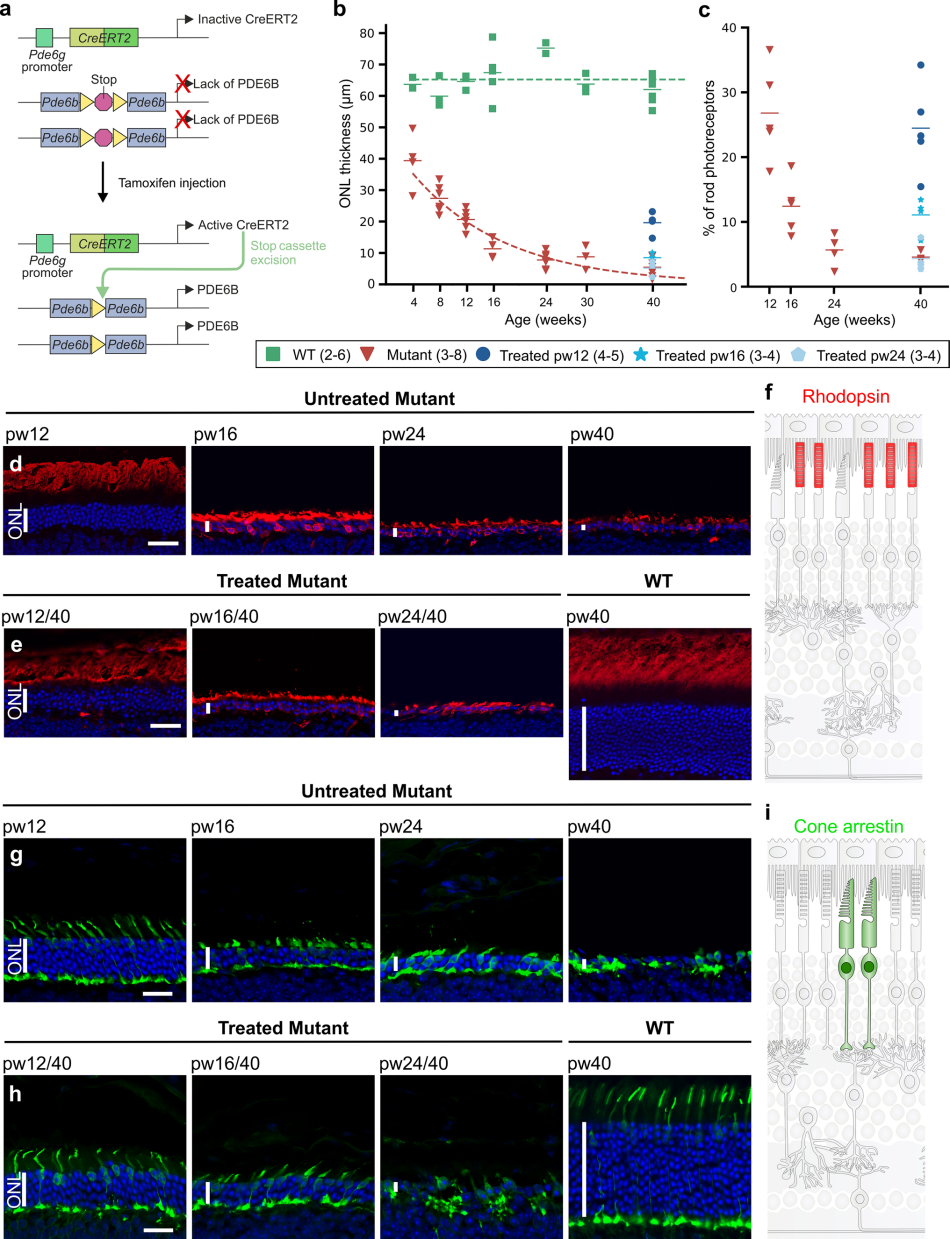
Photoreceptor degeneration halted by treatment at 12 or 16 weeks of age, but not at 24 weeks.** a** Genomic representation of our *Pde6b*^*STOP/STOP*^*, Pde6g*^*CreERT2/*+^ knockin mouse with a transcriptional stop cassette inserted into the intron 1 of the *Pde6b* gene, and tamoxifen-inducible CreERT2 recombinase expression under the control of rod-specific *Pde6g* promoter. Tamoxifen injection activates CreERT2 recombinase, which splices out the stop cassette, leading to *Pde6b* expression. **b** ONL thickness of *Pde6b*^*STOP/STOP*^ (mutant) and *Pde6b*^*STOP/*+^ (WT) mice from 4 to 40 weeks of age, as well as mutants treated at 12, 16, and 24 weeks of age and analyzed at pw40. Horizontal bars, mean ONL thickness. **c** Percent of rods in mutant mice at 12, 16, 24, and 40 weeks of age and in mice that were treated at 12, 16 and 24 weeks and analyzed at 40 weeks of age (relative to 40-week-old WT retinas). Horizontal bars, mean rod percentage. Representative images of retinal sections immunostained with **d**, **e** anti-rhodopsin antibody, **g**, **h** anti-cone arrestin and counterstained with Hoechst. **d, g** Untreated *Pde6b*^*STOP/STOP*^ mice analyzed at 12, 16, 24, and 40 weeks of age. **e**, **h** *Pde6b*^*STOP/STOP*^ mice injected with tamoxifen at 12, 16, and 24 weeks of age, and then analyzed at 40 weeks (treated). Pde6b^STOP/+^ (WT; not treated) analyzed at 40 weeks of age. Schematic representation of healthy **f** rod outer segments and **i** cone cells. *N* values, provided in legend next to each group and in “Materials and methods”. Vertical white bars in **d**, **e**, **g**, and **h**, ONL. Horizontal scale bars, 30 µm (**d**, **e**) and 20 µm (**g**, **h**)

In a previous publication (Koch et al., 2015), we used our genetically engineered retinitis pigmentosa (RP) gene therapy mouse model *Pde6b*^*STOP/H620Q*^, *Pde6g*^*CreERT2/*+^ to study photoreceptor rescue. These *Pde6b*^*STOP/H620Q*^ mice contain a floxed stop cassette on one allele and point mutation H620Q on the other. Here, we used a slightly modified model, *Pde6b*^*STOP/STOP*^*, Pde6g*^*CreERT2/*+^ (*Pde6b*^*STOP/STOP*^). These mice contain a floxed stop cassette in both *Pde6b* alleles that prevents gene expression in the absence of Cre recombinase activity. After tamoxifen injection (referred to here as “treatment”), *Pde6g*^*CreERT2*^ recombinase is activated, the stop cassette removed, and PDE6B expressed. *Pde6b*^*STOP/*+^, *Pde6g*^*CreERT2/*+^ mice (WT) served as controls (Fig. 1a). In this study, we had two goals: first, to test the ability of genetic rescue therapy, administered at late disease stages, to halt and/or reverse the progressive loss of photoreceptor structure and function in RP retinas; second, to understand, at a cellular level, how the retina remodels to adapt to RP disease progression, and how our gene therapy model impacts that remodeling.

In untreated *Pde6b*^*STOP/STOP*^ mice, outer nuclear layer (ONL) thickness had already decreased by 36% at 4 weeks of age, and by 67%, 82% and 87% at 12, 16, and 24 weeks, respectively (Fig. 1b; relative to 40-week-old WT). Rod photoreceptor counts in untreated Pde6b mice were 27%, 12% and 6% at week 12, 16 and 24, respectively. At 40 weeks of age, 5% of rod photoreceptors remained in untreated animals and 25%, 11% and 4% in animals treated at 12, 16 and 24 weeks of age, respectively (Fig. 1c; Table 4). In *Pde6b*^*STOP/STOP*^ mice treated at 12 or 16 weeks of age and analyzed at 40 weeks, ONL degeneration was halted at the disease stage at which treatment was administered (Fig. 1b, d-h; Table 5). In contrast, treatment at 24 weeks did not appear to impact degeneration in the ONL. To analyze rod outer segments, we immunolabeled sections with rhodopsin antibody. In untreated *Pde6b*^*STOP/STOP*^ mice, rod outer segment length gradually decreases over time, and rhodopsin redistributes from outer segments to inner segments and photoreceptor cell bodies. In addition, we observed rhodopsin-positive neurites extending into the inner plexiform layer (Fig. 1d). In *Pde6b*^*STOP/STOP*^ mice treated at 12 or 16 weeks of age, rod outer segments were preserved. In mice treated at 24 weeks of age, rhodopsin redistribution and neurite extension occurred (Fig. 1e). In RP, rod degeneration leads to secondary degeneration of cones. To assess cone response to treatment, retinal sections were immunolabeled with anti-cone arrestin antibody. In untreated *Pde6b*^*STOP/STOP*^ mice, cones progressively degenerate over time (Fig. 1g). In *Pde6b*^*STOP/STOP*^ mice treated at 12 or 16 weeks of age, further cone degeneration was prevented (Fig. 1h). However, in *Pde6b*^*STOP/STOP*^ mice treated at 24 weeks of age, cone degeneration continued.

**Table 4:** Number of rod photoreceptors

Untreated		Treated	
Age at analysis (weeks)	Rod number (mean ± SEM; %)^+^	Age at treatment/ analysis (weeks)	Rod number (mean ± SEM; %)^+^
12	26.8 ± 3.2	12/40	24.5 ± 3.1
16	12.4 ± 1.9	16/40	11.1 ± 1.4
24	5.6 ± 1.3	24/40	4.3 ± 1.1
40	4.6 ± 0.4		

^ +^Relative to the rod number of WT at 40 weeks

**Table 5:** ONL thickness in mutant and treated mice

Untreated		Treated	
Age at analysis (weeks)	ONL thickness (mean ± SEM; µm)	Age at treatment/ analysis (weeks)	ONL thickness (mean ± SEM; µm)
12	20.6 ± 1.3	12/40	19.6 ± 1.8
16	11.3 ± 1.6	16/40	8.5 ± 0.9
24	7.8 ± 0.9	24/40	5.2 ± 1.4
40	5.4 ± 1.0		

These findings are supported by our ERG data (Suppl. Figure 1). B-wave amplitudes (i.e., inner retinal responses) driven by rods and cones (Suppl. Figure 1b, d) or cones alone (Suppl. Figure 1c, e) of *Pde6b*^*STOP/STOP*^ retinas were partially rescued by treatment at either pw12 or pw16 (vs untreated *Pde6b*^*STOP/STOP*^), but not at pw24. ERG is insufficiently sensitive to detect a-wave (i.e., photoreceptor) amplitude recovery (Suppl. Figure 1a, d).

These data show for the first time that gene therapy can halt photoreceptor loss in RP retinas at late disease stages (16 weeks of age and possibly later). In addition, the data suggest that in our RP mice, there is a point of no return for photoreceptor rescue, and it is between 16 and 24 weeks of age.

### Visual function completely rescued by treatment at 16 weeks of age or earlier, but not by treatment at 24 weeks

To test our gene therapy model in a more physiologically relevant, “patient-centered” function, we utilized the Morris water maze test. In this vision-guided behavior assay, mice use visual cues to orient themselves and locate a hidden submerged platform, and the time that it takes them to do so is measured as “escape latency.”

**Fig. 2:**
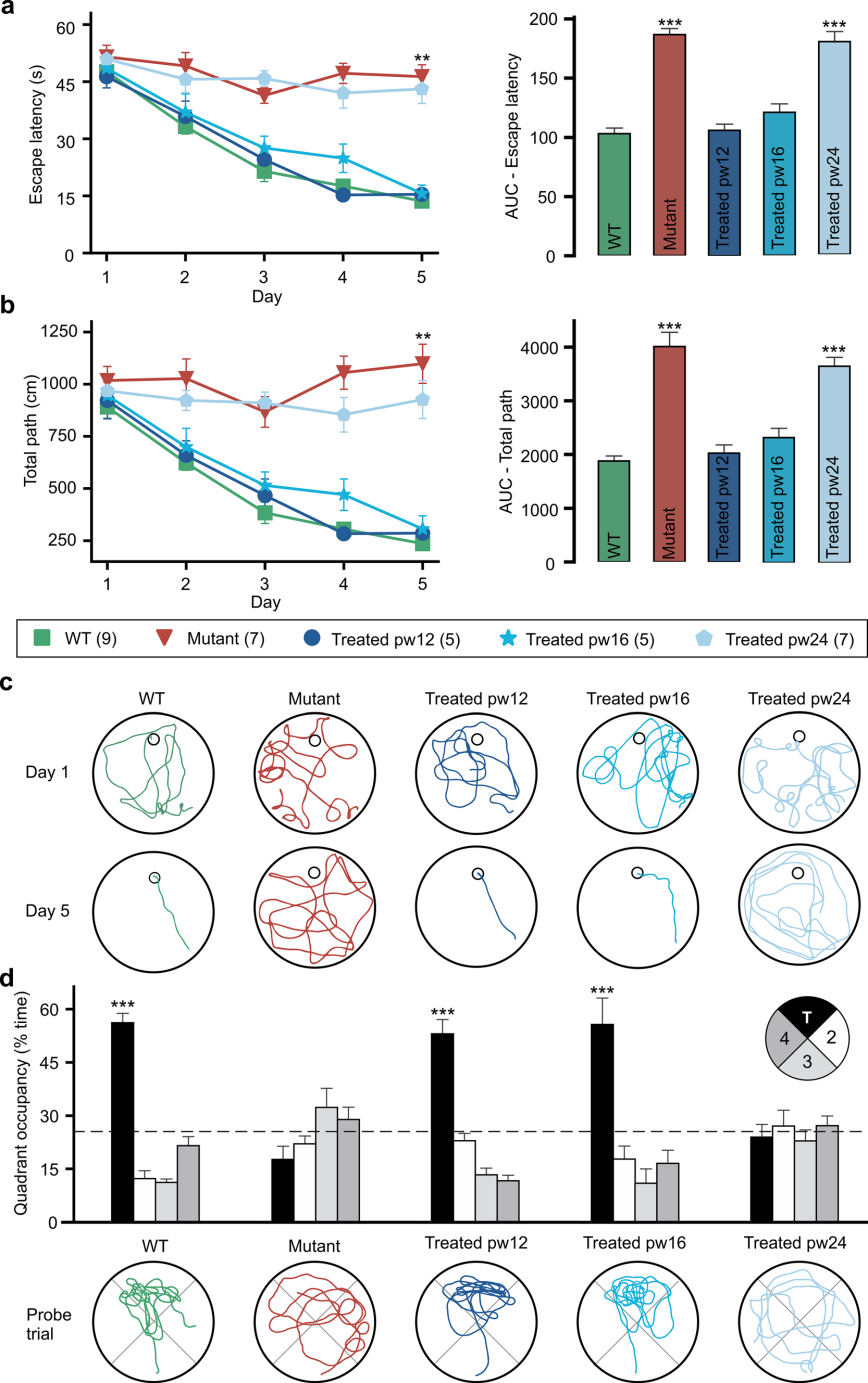
Rescue of vision after treatment at 12 or 16 weeks of age, but not at 24 weeks. *Pde6b*^*STOP/STOP*^ mice were treated at 12, 16 or 24 weeks of age, and then subjected to the Morris water maze behavioral test (under dim-light conditions) over 5 consecutive days at 40 weeks of age (or 30 weeks for pw12-treated mice). **a** Escape latency (time to find the hidden platform) and **b** total path (from starting point to platform)—presented as the mean ± SEM on the left and the area under the curve (AUC) on the right. Asterisks, significance for mutant and treated pw24 vs WT, treated pw12, and pw16. **c** Representative swimming trajectories for days 1 and 5. **d** Probe trial; platform was removed from the north target (T) quadrant, and time spent in each quadrant measured. Data presented as mean percentage (± SEM) of time spent in each quadrant; dashed line, chance level (25%). Representative swimming trajectories for the probe trial are shown at the bottom. Asterisks, significance for T vs other quadrants. Tukey’s test for multiple comparisons. **P ≤ .01; *** *P* ≤ .001. *N* values indicated in legend next to each group

On day 1 of 5, there was no significant difference in escape latency between any of the five groups. Over the 5 days, escape latency remained around 50 s for untreated *Pde6b*^*STOP/STOP*^ mice (Fig. 2a), but decreased to 14 s for WT mice. Like the WT, escape latency in *Pde6b*^*STOP/STOP*^ mice treated at 12 or 16 weeks decreased over time, such that on day 5 there was no significant difference between these two treatment groups and WT. In contrast, *Pde6b*^*STOP/STOP*^ mice treated at 24 weeks of age, like untreated mutants, showed no significant decrease over time. We next compared escape latency over the entire 5-day training period by analyzing the area under the curve (AUC) (Fig. 2a; right panel). The AUC data for WT and *Pde6b*^*STOP/STOP*^ mice treated at weeks 12 or 16 were not significantly different and were all dramatically lower than either untreated or week-24-treated *Pde6b*^*STOP/STOP*^ mice.

We also analyzed path length from the starting point to the platform and found the results to be strikingly similar to escape latency. Mean path length progressively decreased over the 5 days in WT mice and *Pde6b*^*STOP/STOP*^ mice treated at 12 or 16 weeks of age (Fig. 2b). In contrast, path lengths in untreated *Pde6b*^*STOP/STOP*^ mice and mice treated at 24 weeks remained high. The total path length over the 5-day training period (AUC) was significantly different for untreated *Pde6b*^*STOP/STOP*^ mice vs WT or *Pde6b*^*STOP/STOP*^ mice treated at 12 or 16 weeks of age (Fig. 2b; right panel). *Pde6b*^*STOP/STOP*^ mice treated at 24 weeks of age vs untreated mice were not significantly different. These findings are illustrated by representative swim paths from days 1 and 5 for all five groups (Fig. 2c).

Finally, we performed a probe trial, in which the platform (located in the “target quadrant”) was removed, mice given 60 s to search for it, and the time spent in each quadrant measured (Fig. 2d). WT mice and *Pde6b*^*STOP/STOP*^ mice treated at 12 or 16 weeks spent 56%, 53% and 55% (respectively) of the time in the target quadrant. In contrast, for *Pde6b*^*STOP/STOP*^ mice that were either untreated or treated at 24 weeks of age the numbers were 18% and 24%, respectively. These findings are also illustrated by representative swim paths (Fig. 2d; bottom panel).

### RPE remodeling continues after treatment at pw16 and pw24

**Fig. 3:**
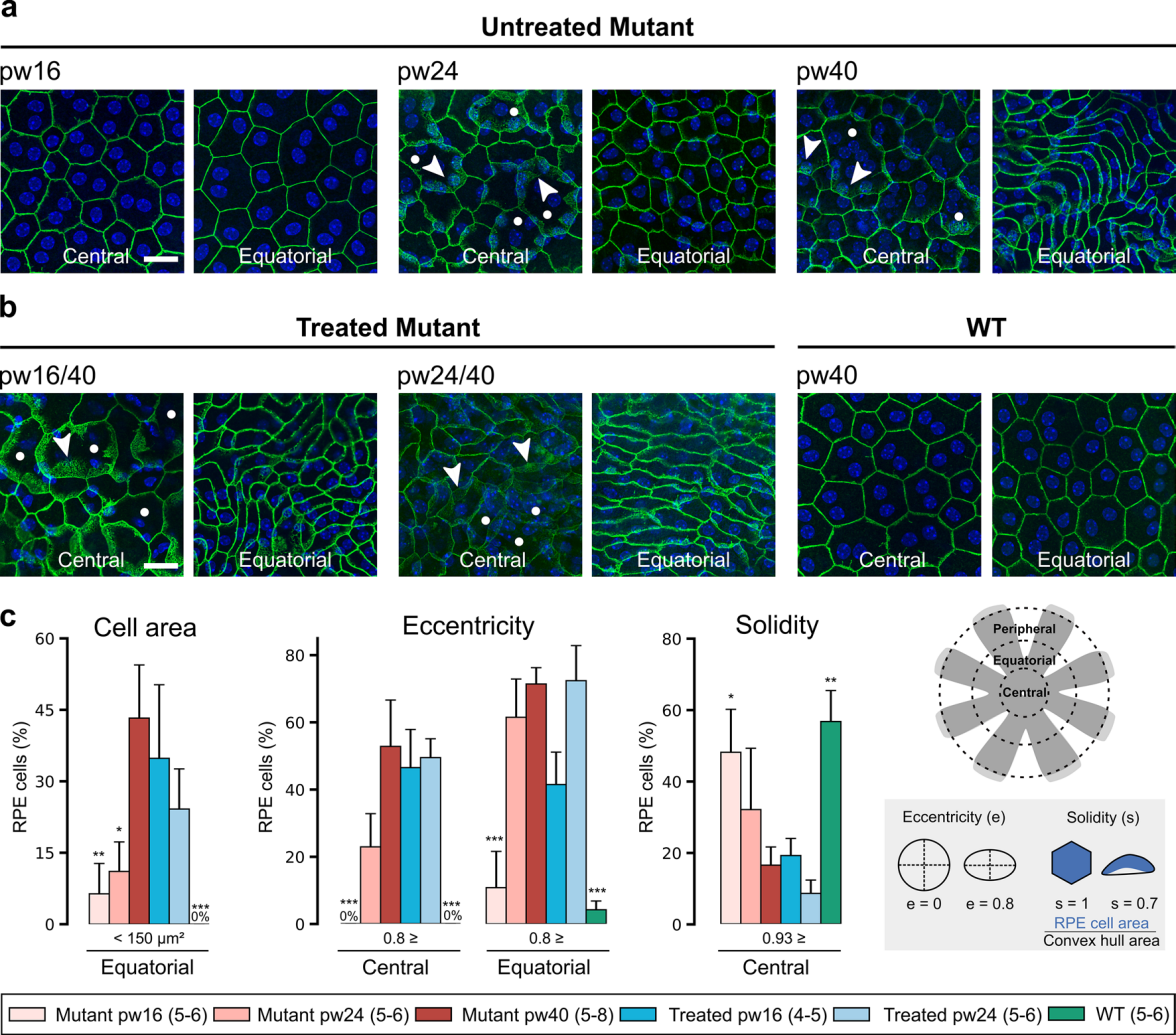
RPE remodeling continues after treatment 16 or 24 weeks of age. Untreated *Pde6b*^*STOP/STOP*^ mice were analyzed at 16, 24 or 40 weeks of age. Pde6b^STOP/+^ (WT) mice and *Pde6b*^*STOP/STOP*^ mice treated at 16 and 24 weeks of age were all analyzed at 40 weeks of age. RPE–choroid–sclera flat mounts were isolated and labeled with anti-β-catenin antibody (green) to visualize a component of adherens junctions. **a**, **b** Images taken in central and equatorial region of RPE flat mounts. Arrowheads, diffuse β-catenin labeling in the cytoplasm; white dots, enlarged RPE cells. Scale bars, 20 µm. **c**, **d** RPE cell features quantified in central and equatorial regions: cell area, eccentricity and solidity. Data represent mean percentage ± SEM; Tukey’s test for multiple comparisons. *P ≤ .05; ** *P* ≤ .01; *** *P* ≤ .001 (all in comparison to mutant). *N* values, provided in legend next to each group and in “Materials and methods”

Photoreceptor death in RP drives changes in the RPE monolayer [4, 30]. To characterize these changes and study their response to treatment, RPE cells were delineated by immunolabeling flat-mounted RPE–choroid–sclera with antibody against the cell-adhesion protein β-catenin. In pw40 WT and pw16 untreated *Pde6b*^*STOP/STOP*^ mice, RPE cells had a polygonal (mostly hexagonal) shape and somewhat uniform size, and β-catenin immunolabeling was located solely at cell–cell contacts (Fig. 3a, b). In central RPE of untreated mice at pw24, cell size was more irregular—with both abnormally large and small cells visible (Fig. 3a), and β-catenin immunolabeling was detectable in the cytoplasm (Fig. 3a; arrowheads). By pw40, RPE cells in the equatorial region exhibited strikingly elongated cells and diffuse cytoplasmic β-catenin (Fig. 3a). In the periphery up to pw40, RPE cells show only minor morphological changes (Fig. S2). In *Pde6b*^*STOP/STOP*^ mice treated at either 16 or 24 weeks, this disease-driven remodeling of RPE cells appeared to continue unabated (Fig. 3b).

To quantify these observations, we analyzed cell area (size), cell eccentricity (shape/elongation) and solidity (proportion of the RPE cell area filling a best-fit convex envelope) (Fig. 3c). In WT mice, no RPE cells were smaller than 150 µm^2^ in the equatorial region, there were no elongated cells centrally and only very few equatorially, and most cells had a solidity ≥ 0.93 (Fig. 3c). Quantifications in untreated *Pde6b*^*STOP/STOP*^ mice were similar to WT at 16 weeks, but became increasingly distinct, so that by pw40, 43% of RPE cells were smaller than 150 µm^2^ (equatorial), which is significantly different from both 24-week-old untreated animals and WT, 53% and 71% of RPE cells (central and equatorial regions, respectively) had an eccentricity ≥ 0.8, and significantly fewer RPE cells (vs WT) had a solidity ≥ 0.93 (Fig. 3c). In *Pde6b*^*STOP/STOP*^ mice treated at 16 or 24 weeks (vs 40-week untreated mutant), cell area, eccentricity and solidity were not statistically different (Fig. 3c).

### Remodeling of inner retinal cells halted by treatment at 16 weeks of age or earlier, but not at 24 weeks

Cells in the inner nuclear layer remodel in response to photoreceptor degeneration [30]. To evaluate whether these changes can be halted or even reversed by treatment, we analyzed morphological changes in horizontal and bipolar cells in retinal sections from treated and untreated *Pde6b*^*STOP/STOP*^ mice.

**Fig. 4:**
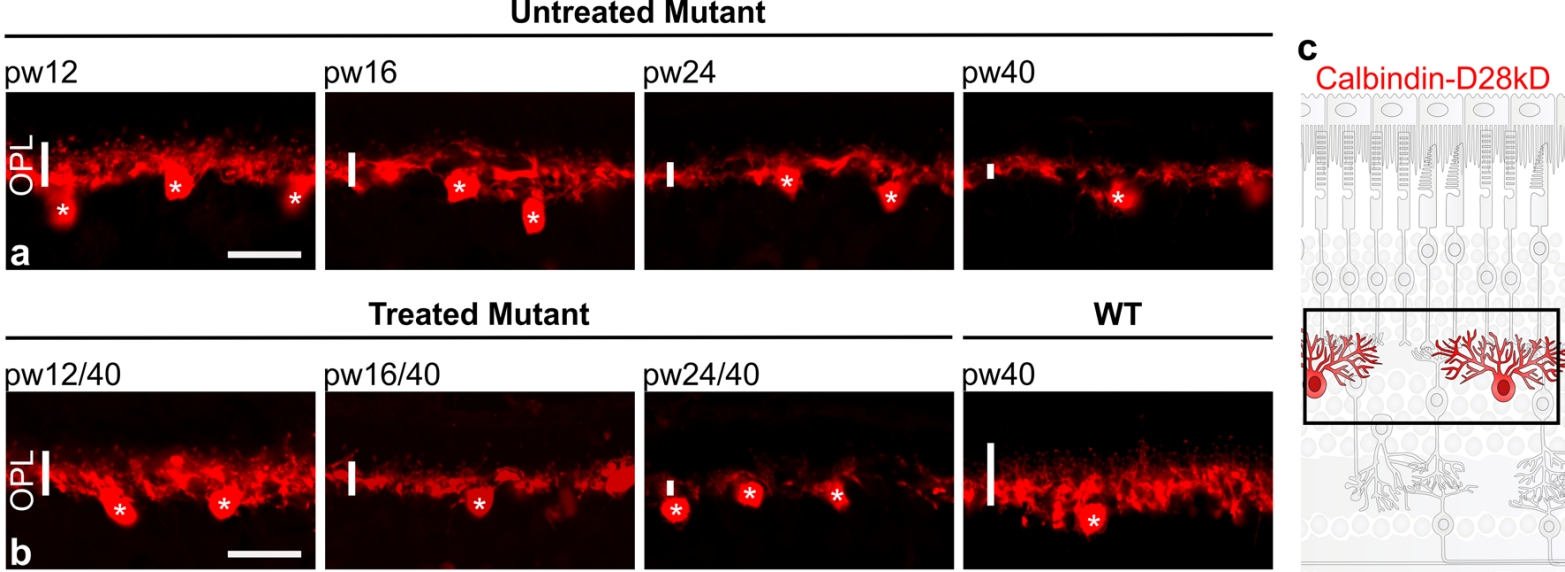
Horizontal cell remodeling halted by treatment at 12 and 16 weeks of age, but not at 24 weeks. *Pde6b*^*STOP/STOP*^ mice were treated (or not) at pw12, pw16 or pw24, and analyzed at pw40. *Pde6b*^*STOP/*+^ mice (WT) were 40 weeks of age. Retinal sections were labeled with anti-calbindin-D28kD antibody to visualize horizontal cells, including their processes in the OPL (vertical white bars). Retinal sections from **a** untreated *Pde6b*^*STOP/STOP*^ mice, and **b** treated *Pde6b*^*STOP/STOP*^ and *Pde6b*^*STOP/*+^ (WT) mice. Asterisks indicate horizontal cell bodies. Scale bars, 20 µm. **c** Schematic representation of healthy horizontal cells

Horizontal cells were visualized using calbindin-D28kD antibody. Immunolabeled WT retinas revealed a dense meshwork of dendrites with tiny puncta in the outer plexiform layer (OPL) (Fig. 4b; right-most panel). In *Pde6b*^*STOP/STOP*^ mice, the dendritic processes gradually retracted and the puncta decreased as the disease progressed, forming only a very porous meshwork by week 40 (Fig. 4a). In mice treated at 12 or 16 weeks of age, the density of dendritic processes of the horizontal cells appeared to be preserved. In contrast, in retinas of mice treated at week 24, retraction of horizontal cell dendrites was not halted (Fig. 4b; left panels).

**Fig. 5:**
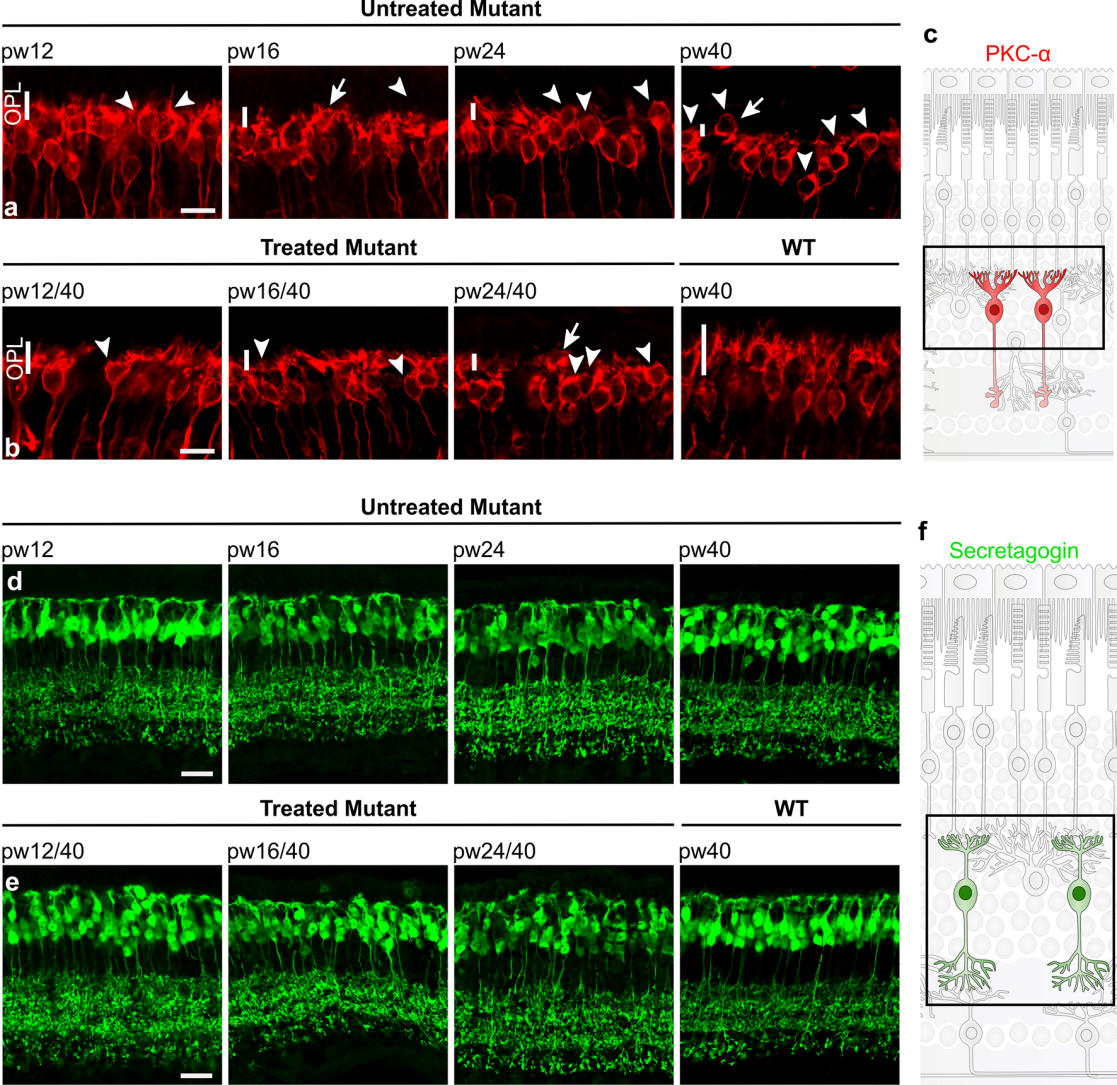
Bipolar cell remodeling halted by treatment at 12 and 16 weeks of age, but not at 24 weeks. *Pde6b*^*STOP/STOP*^ mice were treated (or not) at pw12, pw16 or pw24, and analyzed at pw40. *Pde6b*^*STOP/*+^ mice (WT) were 40 weeks of age. Retinal sections were labeled with **a**, **b** anti-PKCα antibody to visualize rod bipolar cells, and **d**, **e** anti-secretagogin antibody to visualize cone bipolar cells particularly their processes in the OPL. Sections from **a**, **d** untreated *Pde6b*^*STOP/STOP*^ mice, and **b**, **e** treated *Pde6b*^*STOP/STOP*^ and *Pde6b*^*STOP/*+^ (WT) mice. Vertical white bars in **a** and **b**, rod bipolar dendrites in the OPL; arrowheads, rod bipolar cells having aberrant dendrites; arrows, ectopic cell body. Schematic representation of healthy **c** rod bipolar cells and **f** cone bipolar cells. Scale bars, 10 µm (**a**, **b**) and 20 µm (**d**, **e**)

In WT retinas, rod bipolar cells had dendritic arborization in the OPL with numerous dendrites irradiating from the cell (Fig. 5b; right-most panels). In untreated *Pde6b*^*STOP/STOP*^ retinas, these dendrites progressively retract (Fig. 5a), so that by 40 weeks of age the OPL was devoid of almost all rod bipolar cell dendritic processes; in addition, rod bipolar cell bodies were sometimes seen in the ONL. In *Pde6b*^*STOP/STOP*^ mice treated at 12 or 16 weeks of age, retraction of the dendrites was halted (Fig. 5b). However, in *Pde6b*^*STOP/STOP*^ mice treated at 24 weeks of age, rod bipolar cell dendrite retraction seemed to continue.

Lastly, retinal sections were stained with an antibody against secretagogin, which recognizes most types of cone bipolar cells [31]. In WT retinas, cone bipolar major dendritic branches emerge from the soma and split into minor branches in the OPL and their axon terminals are stratified in the inner plexiform layer (Fig. 5e; right-most panel). In *Pde6b*^*STOP/STOP*^ mice, cone bipolar cells did not dramatically remodel (Fig. 5d), reflecting the later onset of cone (vs rod) degeneration. By week 40, some cone bipolar cells had retracted their dendrites and migrated toward the ONL. These morphological changes were halted in mice treated at 12 or 16 weeks of age. However, treatment at 24 weeks did not stop the remodeling as some irregular arrangements of the cone bipolar cell bodies and dendrites were observed (Fig. 5e).

### Blood vessel remodeling is partially impacted/affected by treatment

**Fig. 6:**
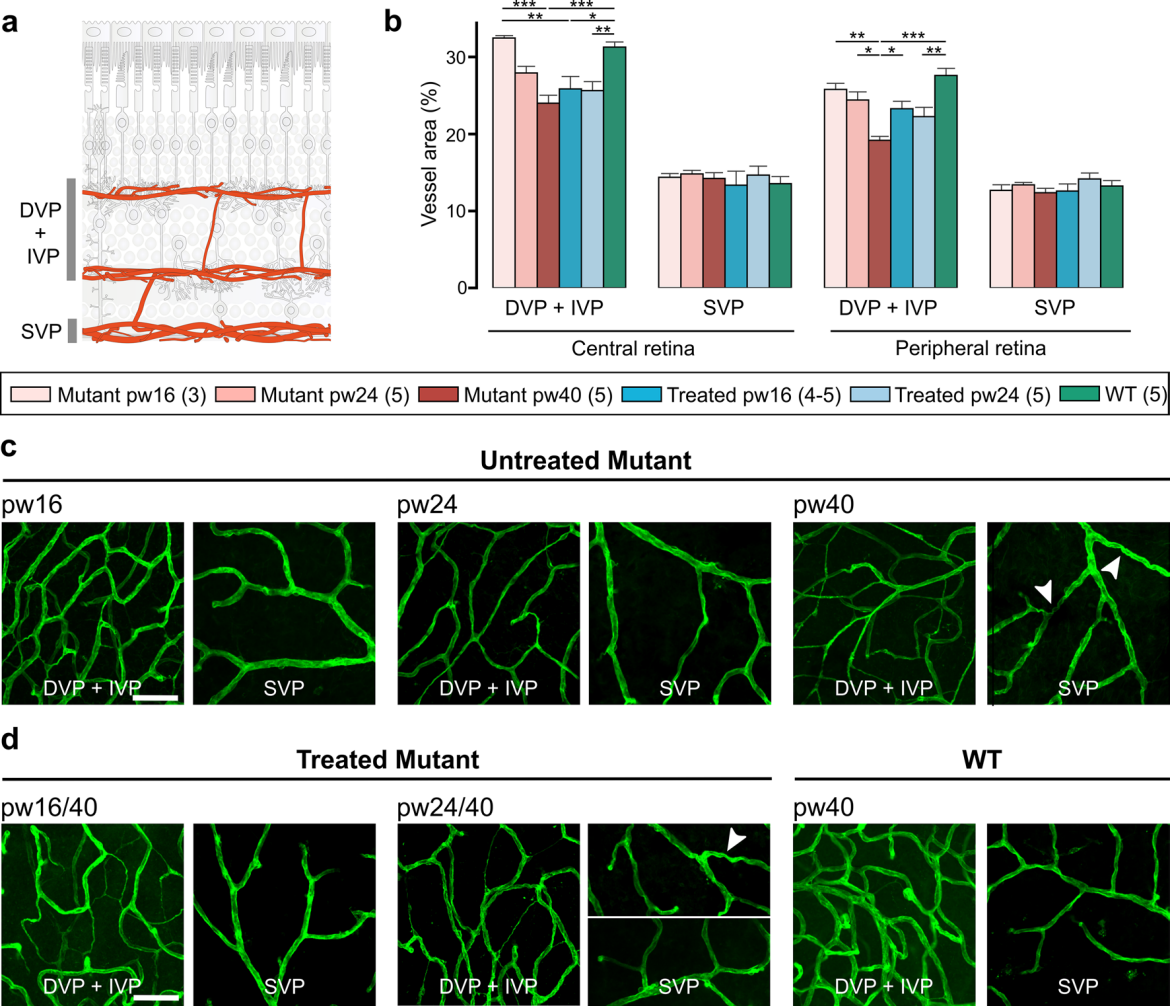
Central blood vessel area decrease cannot be prevented by treatment at 16 weeks of age. Untreated *Pde6b*^*STOP/STOP*^ mice were analyzed at 16, 24 or 40 weeks of age. Pde6b^STOP/+^ (WT) mice and *Pde6b*^*STOP/STOP*^ mice treated at 16 and 24 weeks of age were all analyzed at 40 weeks of age. Retinas were stained with isolectin GS-IB4, and vascular areas determined using AngioTool software. **a** Schematic representation of the three retinal vascular plexi. **b** Area quantification of DVP + IVP (merged in ImageJ) and SVP in central and peripheral retina. Data presented as mean ± SEM; Tukey’s test for multiple comparisons. *P ≤ .05; ** *P* ≤ .01; *** *P* ≤ .001. *N* values provided in legend next to the groups. **c**,** d** Representative images. Arrowheads, tortuous vessels. Scale bars, 50 μm

In RP retinas, photoreceptor loss is followed by reduced retinal vessel density [32–35]. To better understand vascular pathology in treated and untreated RP retina, we stained whole-mounted retinas with isolectin GS-IB4, an endothelial cell marker, to quantify the area of the three-layered retinal vascular network supplying the inner retina: superficial, intermediate and deep vascular plexi (SVP, IVP and DVP) (Fig. 6a; schematic). In treated and untreated and WT mice, the SVP area in peripheral and central retina remained the same. In contrast, IVP + DVP area was decreased in both central and peripheral retina of untreated mutants at 40 weeks (vs WT) (Fig. 6b)—due, at least in part, to a thinning of the vessel diameter (Fig. 6c, d). Treatment at 16 weeks of age partially prevented some of this decrease in peripheral retina but not in central retina (Fig. 6b, d). In *Pde6b*^*STOP/STOP*^ mice treated at 24 weeks, blood vessel degeneration continued unabated (Fig. 6b, d). Tortuous vessels were seen in untreated mice at 40 weeks of age and in some mice treated at 24 weeks (Fig. 6c, d).

**Fig. 7:**
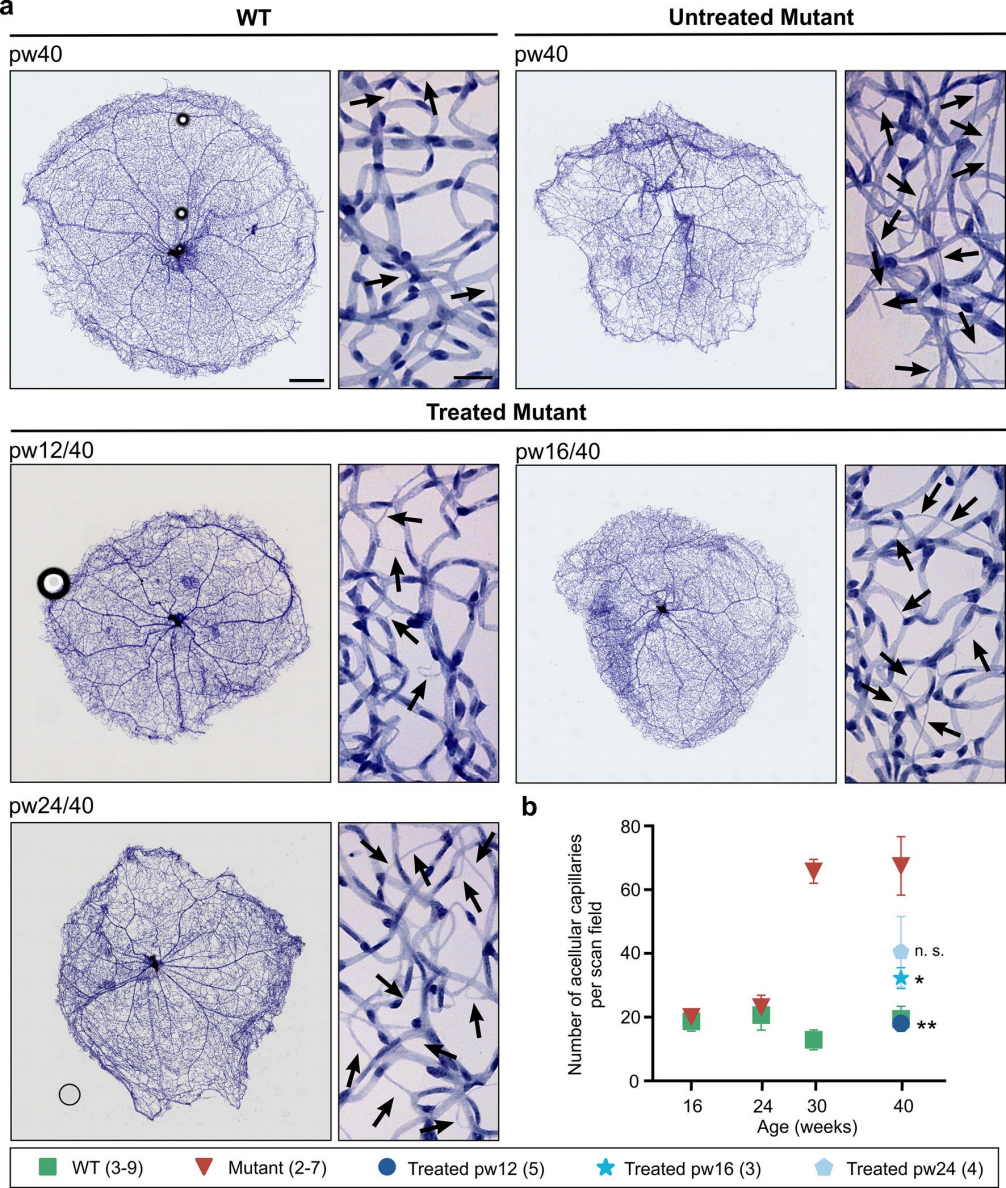
Quantification of acellular capillaries. Untreated *Pde6b*^*STOP/STOP*^ (mutant) and *Pde6b*^*STOP*/+^ (WT) mice were analyzed at 16, 24, 30, and 40 weeks of age. *Pde6b*^*STOP/STOP*^ mutant mice were treated at 12, 16 or 24 weeks of age, and all analyzed at 40 weeks of age. Whole-mounted retinas were trypsin digested and stained with hematoxylin–eosin. **a** Representative images of whole retinal vasculature and higher-magnification images—all at 40 weeks of age. Black arrows, acellular capillaries; scale bars, 500 µm (whole retina/low mag) and 25 µm (high mag). **b** Mean number of acellular capillaries per scan field (± SEM); *t* test comparing treated mutants vs 40-week-old untreated mutant; n.s. not significant; *P ≤ .05; ** *P* ≤ .01. *N* values are provided in the legend next to the groups and in detail in “Materials and methods”

When endothelial cells die, the result is acellular capillaries, empty basement membrane sleeves with no endothelial cell nuclei along their length [36]. The measurement of these non-perfused acellular capillaries is an important marker for monitoring progression of RP and treatment response. To assess the formation of acellular capillaries, whole retinas from WT and untreated *Pde6b*^*STOP/STOP*^ mice were analyzed at 16, 24, 30, and 40 weeks of age, and from treated *Pde6b*^*STOP/STOP*^ mice at 40 weeks (Fig. 7). In WT retinas, a small number of acellular capillaries are present and their numbers remain steady up to 40 weeks of age. In untreated *Pde6b*^*STOP/STOP*^ retinas (vs WT), the numbers of acellular capillaries were not significantly different up to 24 weeks of age, but was dramatically increased at 30 and 40 weeks of age (*P* = 0.002 and *P* = 0.0001, respectively) (Fig. 7b). In addition, whole retinal vasculature of *Pde6b*^*STOP/STOP*^ mice (vs WT) at pw40 is disrupted, and there are avascular spots that seem diffusely distributed (Fig. 7a). After treatment at 12 weeks of age, the number of acellular capillaries was not significantly different from WT (*P* = 0.99) (Fig. 7b). In mice treated at 16 weeks (vs WT), we observed a small increase in the number of acellular capillaries, which was not statistically significant (*P* = 0.8). The number of acellular capillaries was significantly different compared to mutant pw40 (*P* ≤ 0.05). For retinas treated at 24 weeks, the mean number of acellular capillaries was not significant compared to mutant pw40 (*P* = 0.08).

## Discussion

When RP patients seek medical help, their vision is typically already impaired [2]. It is therefore important that RP therapies provide sustainable rescue of retinal function at even mid-to-late disease stages. In a previous publication, we used one of our genetically engineered RP gene therapy mouse models to demonstrate sustained rescue of photoreceptor structure (ONL thickness) and retinal function (ERG) at early (2 and 4 weeks of age) and mid-disease stages (8 weeks of age) [3]. In this current study, we tested whether our genetic rescue can impact visually guided behavior, and whether rescue can be extended to late disease stages (12, 16 and 24 weeks of age). Additionally, we performed an extensive morphological analysis of treated, remodeled RP retina, to better understand the structural underpinnings of the RP therapeutic efficacy.

### Successful therapeutic rescue of RP mice extends to late disease stages

Strikingly, our data demonstrate that treatment at 12 or 16 weeks of age leads to complete and long-term rescue of the visually guided behavior (Morris water maze). By all measures (escape latency, path length, area under the curve and probe trial), the rescued behavior at these mid and late time points was indistinguishable from that of wild type. On the other hand, the visually guided behavior of RP mice treated at 24 weeks of age was generally indistinguishable from that of untreated RP mice. In addition, our data show that treatment at 12 or 16 weeks of age (but not at 24 weeks) halts the progressive loss of photoreceptors (ONL thickness, rod counts, or analysis of anti-arrestin labeled cones) (Fig. 1) and retinal function (ERGs) (Fig. S1).

These data show that even in retinas with only 12% or 27% of rod photoreceptors remaining (16 and 12 weeks of age, respectively), mice exhibit complete rescue of a visually guided complex behavior. This argues for retinal remodeling (neurons, supporting cells and vasculature) contributing a role in driving the observed treatment efficacy. In contrast, we did not observe rescue of vision in mice treated at 24 weeks of age (with 6% remaining rods at the time of treatment). We therefore postulate that by 24 weeks of age, the remaining rods are irreversibly damaged and are in an abnormal mutant steady state [28]. In addition, by 24 weeks, the retina may no longer be capable of sufficiently remodeling/compensating.

### Restoration of dendritic length of inner retinal neurons is not required for complete rescue of visually guided behavior

In response to photoreceptor degeneration in RP, rod bipolar and horizontal cells are deafferented and, as a consequence, retract their dendrites [9]. Despite this degradative remodeling, rod bipolar cells [14] and rod photoreceptor cells [37, 38] show increased sensitivity. We also observed dendrite truncation in our *Pde6b*^*STOP/STOP*^ RP mouse model (Figs. 4, 5). Inner retinal cell morphology has been studied following rod photoreceptor gene therapy at early disease stages [39–41]. Our study is the first to treat multiple late disease stages, and also to analyze whether bipolar and horizontal cell remodeling is halted at the stage at which treatment was administered. Our data show that retraction of dendrites from horizontal and rod bipolar cells is halted by treatment at 12 or 16 weeks of age, but not at 24 weeks (Figs. 4b, 5b). Thus, our visually guided behavior data show that dim-light (rod-dependent) vision persists up to a late disease stages (pw12 and 16; Fig. 2), when rod bipolar cell dendrites are already severely truncated, suggesting that it is not necessary to restore dendritic length to achieve complete rescue of visually guided behaviors.

While retinal and other central nervous system dendrites retract and expand during development [42, 43], studies of dendrite-regenerative capacity in adult mammals are limited. In medial prefrontal cortex, stress-induced dendritic retraction is followed by complete reversal only if the stressor and recovery periods are each less than 3 weeks [44] demonstrating that some dendrites possess the capability to regrow after relatively brief periods of retraction. In adult animals, regeneration of dendrites was observed following administration of a neurite growth-promoting factor [45].

### Ongoing potentially constructive RPE and retinal blood vessel remodeling despite treatment

Previous studies demonstrated that in RP retinas, the RPE responds to photoreceptor degeneration in a variety of ways [4, 46]. In line with these findings, we detected progressive cell size irregularity and loss of honeycomb structure (Fig. 3a). We also discovered a shift in β-catenin from the plasma membrane to the cytoplasm (Fig. 3a; arrows), which is associated with non-polarized, invasive and migratory RPE cells in inherited retinal degenerations [47]. Unlike photoreceptor degeneration, photoreceptor functional loss and inner retinal remodeling, this RPE remodeling was not halted by treatment at 16 or 24 weeks of age (Fig. 3b, d). This persistent, potentially constructive remodeling could be driven by the diminished numbers of photoreceptors and shortened outer segments—both of which would lead to reduced photoreceptor light absorption and oxygen consumption and, thus, chronic oxidative stress in the retina. In addition, since RPE metabolism depends on photoreceptors [48, 49], decreased numbers of photoreceptors might lead to RPE starvation. It has been shown that overexpression of NRF2, a transcription factor that regulates the response to oxidative stress, protects RPE in RP mice [46]. Thus, the photoreceptor pathology might lead to the death of some RPE cells, and this degradative remodeling, in turn, may trigger constructive remodeling in the remaining RPE cells. In RP retinas, RPE cells enlarge and/or migrate toward lesion sites, perhaps to preserve an overall functioning RPE monolayer and maintain homeostasis [24]. In fact, enlarged/remodeled RPE cells do not impact visually guided behavior in our mice aged 40 weeks and treated at 16 weeks of age. However, the ongoing RPE remodeling could impair the health of the photoreceptor cells at a greater age in mice or humans.

Vascular damage and dysfunction are frequently associated with neurodegenerative disorders such as dementia and Alzheimer disease [50, 51]. Retinal vascular degeneration has been described in RP patients [32, 33, 52] and animal models [5, 34]. In our untreated RP mice, we observed major degradative remodeling in the retinal vasculature (Fig. 7a, b), including increased numbers of acellular capillaries (Fig. 8). In this study, we show that these changes are prevented by treatment at 12 weeks of age, partially slowed by treatment at 16 weeks, and not impacted by treatment at 24 weeks, probably due to ongoing photoreceptor degeneration and reduced neural activity in the pw24-treated retinas and, thus, reduction in oxygen consumption and metabolic demand. Thus, normal visually guided behavior in our mice treated at 12 or 16 weeks suggests an adequate matching of metabolic needs and blood supply.

## Supplementary Information

Below is the link to the electronic supplementary material.

Supplementary file1 (PDF 707 kb)

## Data Availability

The datasets generated during and/or analyzed during the current study are available from the corresponding author on reasonable request.

## Abbreviations

AUC: Area under the curve
DVP: Deep vascular plexus
ERG: Electroretinography
IVP: Intermediate vascular plexus
ONL: Outer nuclear layer
OPL: Outer plexiform layer
PBS: Phosphate-buffered saline
*Pde6b*: Gene encoding the rod-specific phosphodiesterase 6b
pw: Postnatal week
RP: Retinitis pigmentosa
RPE: Retinal pigment epithelium
SEM: Standard error of mean
SVP: Superficial vascular plexus
WT: Wild type control mice (*Pde6b*^*STOP/*^^+^, *Pde6g*^*CreERT2/*^^+)^
