# Supplementary material for: Late-stage rescue of visually guided behavior in the context of a significantly remodeled retinitis pigmentosa mouse model
Source: Cell Mol Life Sci. 2022 Feb 23;79(3):148. doi: 10.1007/s00018-022-04161-0 (PMC8866266; doi:10.1007/s00018-022-04161-0)
Supplement: Supplementary file 1 — Supplementary file1 (PDF 707 kb) [file 18_2022_4161_MOESM1_ESM.pdf]

Supplementary Information

Late-stage rescue of visually guided behavior in the context of a significantly remodeled retinitis pigmentosa mouse model

Cellular and Molecular Life Sciences

Jacqueline Kajtna[1,2], Stephen H. Tsang[3,4], Susanne F. Koch[1,2*]

[1]Department of Pharmacy, Center for Drug Research, Ludwig-Maximilians-Universität München, Munich, Germany.

[2]Physiological Genomics, BioMedical Center, Ludwig-Maximilians-Universität München, Planegg/Martinsried, Germany.

[3]Jonas Children's Vision Care, Columbia Stem Cell Initiative, Departments of Ophthalmology, Pathology & Cell Biology, Institute of Human Nutrition, Vagelos College of Physicians and Surgeons, Columbia University, New York, New York, 10032, USA.

[4]Edward S. Harkness Eye Institute, New York-Presbyterian Hospital, New York, New York, 10032, USA.

*Correspondence to Susanne F. Koch; Email: susanne.koch@cup.uni-muenchen.de

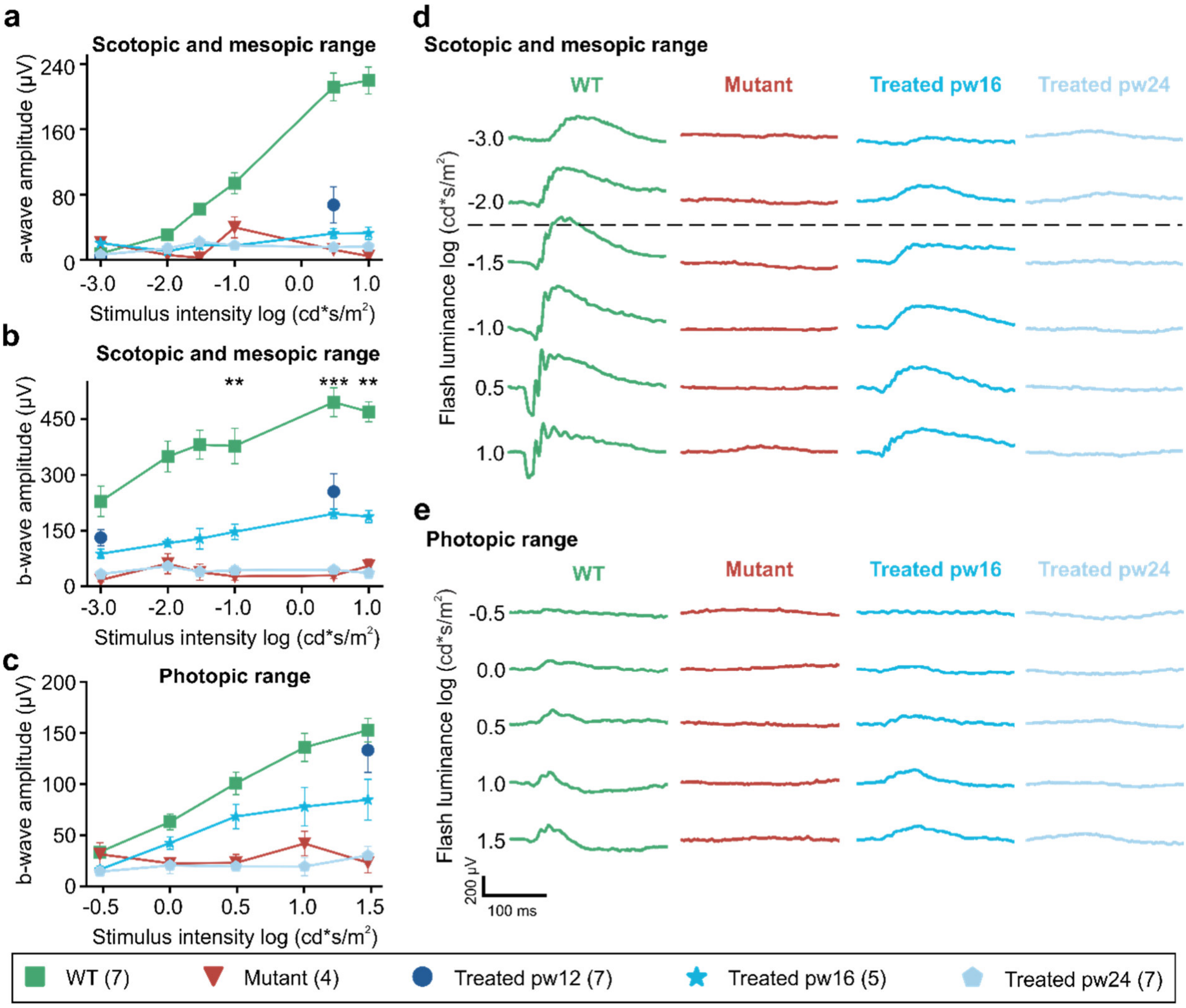

Fig. S1 Rescue of retinal function after treatment at 12 or 16 weeks of age, but not at 24 weeks

Pde6b$^{STOP/STOP}$ mice were treated (or not) at pw12, pw16 or pw24, and full-field single-flash ERGs recorded at pw40. **(a,b)** Scotopic and mesopic a-wave and b-wave amplitudes. **(c)** Photopic b-wave amplitudes. Data represent the mean ± SEM; Tukey's test for multiple comparisons. ** $P \leq .01$; *** $P \leq .001$. Asterisks, significant differences between mutant and treated at pw16. No statistical difference was found between animals treated at pw24 and mutant. N values, indicated in legend next to each group. Representative responses from a single retina in the **(d)** scotopic and mesopic range, and **(e)** in the photopic range. Dashed line, border between scotopic (above) and mesopic ranges (below).

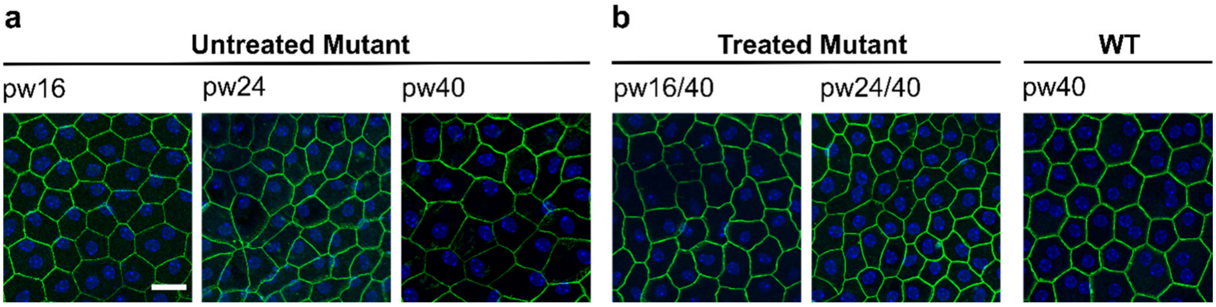

Fig. S2 Minor morphological abnormalities of the peripheral RPE *Pde6b*$^{STOP/STOP}$ mice were sacrificed at 16, 24 or 40 weeks of age. *Pde6b*$^{STOP/+}$ *mice* (WT) *and Pde6b*$^{STOP/STOP}$ mice treated at pw16 and pw24 were all sacrificed at pw40. RPE flat-mounts were labeled with an anti-β-catenin antibody (green) to visualize a component of adherens junctions. **(a)** Representative RPE flat-mounts from untreated *Pde6b*$^{STOP/STOP}$ mice at 16, 24 and 40 weeks of age and **(b)** from 40-week-old treated *Pde6b*$^{STOP/STOP}$ and *Pde6b*$^{STOP/+}$ (WT) mice. Scale bar, 20 µm.
